# Acute Myocardial Infarction Complicated With Ventricular Septal Rupture: Report of Three Cases

**DOI:** 10.4021/cr316e

**Published:** 2014-01-02

**Authors:** Wenrong Su, Shuguang Wang, Jian Wang, Jungang Zhang, Yanbo Chen, Guodong Wang, Aiyuan Zhang

**Affiliations:** a Department of Cardiology, Weifang People’s Hospital, Weifang, Shandong, China

**Keywords:** Ventricular septal rupture, Acute myocardial infarction, Treatment

## Abstract

We reported three cases of ventricular septal rupture (VSR) complicating acute myocardial infarction (AMI), focusing on the causes, diagnosis, treatment and prevention. These three cases were diagnosed based on the findings of electrocardiogram, echocardiogram and blood myocardial markers, and were treated with conservative methods. These three cases were female, and all had history of hypertension and/or diabetes mellitus. In one case with age over 70, AMI was related to percutaneous coronary intervention of left anterior descending branch, and the stenosis of LAD resulted in AMI and subsequently VSR occurred, the patient’s condition worsened rapidly and the patient died after. Only one of the three cases survived the VSR. We concluded that the prognosis of VSR complicating AMI is associated with the causes, age, sex and comorbidities, and the prevention is critically important.

## Introduction

Ventricular septal rupture (VSR) is a severe complication of acute myocardial infarction (AMI), and its morbidity accounts for about 0.5-2% of all AMI. It is often accompanied by severe circulation failure, and 56% of AMI patients with VSR had new murmur and touched tremor in the precordium. In VSR, left-to-right shunt occurs, resulting in insufficient systemic circulation and pulmonary hypertension, then blood circulation is reallocated, which leads to extravasate blood in lungs and digestive tract, the renal blood flow decreases, peripheral circulation deteriorates, and consequently patients suffer shock due to decompensation. The mortality rate in surgical management of VSR is about 30%, and it would be higher in patients with inferior wall myocardial infarction. The mortality in patients with conservative treatment is as high as 80-90%, and the survival is less than 10%. In this report, we review clinical data of three cases of AMI complicated with VSR.

## Case Report

### Case 1

A 76-year-old female presented with nausea and vomiting for 2 days. At admission, the patient was found tachycardic (114 beats/min). Blood pressure was 94/76 mmHg. On work-up, breath sounded roughly, moist rales at the lower of the lungs and a 3/6 systolic murmur was apparent on the apex. Transthoracic echocardiogram (TTE) revealed an estimated ejection fraction (EF) of 41%, and a VSR (diameter 10 mm) with left-to-right shunt, a small amount of tricuspid regurgitation. The 12-lead electrocardiogram showed anteroseptal myocardial infarction. Creatine phosphokinase (CPK) and creatine kinase isoenzyme (CK-MB) levels were 815 U/L (normal value 38-174) and 42 U/L (normal value 1-20 U/L), respectively. Troponin I 59.73 ng/mL (normal value 0-0.04), lactic dehydrogenase, creatinine and urea nitrogen were all increased. The results of hemogram showed white blood cell was 35.60 × 10^9^/L, and blood glucose was 7.4 mmol/L. Urine routines showed red blood cell ++, glucose ++ and ketone body +. The diagnoses were confirmed anteroseptal myocardial infarction with VSR, type 2 diabetes and hypertension. The patient was treated with routine antiplatelet, intravenous heparin, expansion of blood vessels, lowering heart rate, lipid drug and furosemide. Unfortunately, the patient died 24 h after admission.

### Case 2

A 71-year-old female presented with 7 years mild dyspnoea and chest pain, exacerbated for 1 month. Coronary angiography revealed triple vessels inflicted, with 95% stenosis of the middle of the anterior descending branch, 70-80% stenosis of the proximal and 99% stenosis of distal segments of left circumflex artery, 80-90% stenosis of the middle of right coronary artery. The patient was suggested coronary artery bypass graft (CABG). Considering the risk of surgery, her family agreed percutaneous coronary intervention (PCI), and we decided to intervene the left anterior descending artery. During the operation, we found it difficult to pass the guiding wire through the diseased section, and then we discontinued PCI. On day 7 post-intervention, the patient complained of intensified chest pain, which radiated to back, and these symptoms lasted for 1 h. BP was 99/69 mmHg, a new 3/6 systolic murmurs appeared on the apex, CK 176 U/L, CK-MB 34 U/L, troponin I 2.26 ng/mL. Echocardiography showed the left atrium diameter 36 mm, left ventricular diameter 54 mm, right atrium diameter 44 mm, EF 42%, and mobilization was weakened obviously in apex, anterior wall and anteroseptum. There was an abnormal echo in the middle and lower third of muscular section of interventricular septum, with diameter of 9 mm. Calcification and a small amount of regurgitation were seen in aortic valve, and the tricuspid valve had a medium amount of regurgitation, with pulmonary hypertension (PAH) of 52 mmHg. There was a ventricular aneurysm in apex, and VSR. Electrocardiogram showed Q wave and high elevation of ST segment in V1-V4 of precordial leads, and ST segment elevated 0.4 mV in V2-V4. The patient was confirmed diagnosis of AMI complicated with VSR and died 48 h after VSR.

### Case 3

A 61-year-old female complaining of chest pain for 2 months was hospitalized for 1 month in the intensive care unit of a local hospital, prior to admission to our hospital. On work-up, moist rales could be heard in the bottom of the right lung. The 3-4/6 systolic murmurs were prominent in the area of bicuspid valve, tricuspid valve and aortic valve, especially in the area of bicuspid valve. Echocardiography showed EF 45%, and the mobilization of anteroseptum, antetheca and apex decreased. The diameters of all atrial and ventricular chambers were enlarged in various degrees. The wall of anteroseptum and antetheca became thin, about 4 mm. Echo was interrupted in the interventricular septum close to apex, 8 - 11 mm, and with left-to-right shunt. There was a small amount of pericardial effusion. Electrocardiogram showed pathological Q waves in II, III leads, AVF leads, and in V1-V4 of precordial leads. The patient was treated with antiplatelet, anti-coagulation with low molecular heparin, and with dopamine. The patient’s condition improved and was advised to surgical treatment, but she refused and asked for discharge with future follow-up.

## Discussion

### Cardiac rupture

The mortality rate of VSR after AMI is high, and most patients died of shortage of effective treatment. Cardiac rupture (CR) which often is acute always occurs within 1 week after AMI, mainly the VSR. AMI patients with VSR died suddenly because of hemopericardium and pump failure, both resulted from VSR. Some sub-acute VSR patients may survive for a few months. The three cases presented here were all acute anterior myocardial infarction complicated with VSR. Case 1 presented with gastrointestinal symptoms at onset, who was not diagnosed in time and CR occurred, thus the effective treatment was delayed. Case 2 showed triple vessels inflicted in coronary angiography, the plaque was unstablized due to unsuccessful PCI intervention of left anterior descending branch (LAD), subsequently the LAD stenosis resulted in AMI; this was intervention-related AMI, which caused malignant arrhythmia, and finally VSR occurred. Case 3 had been diagnosed hypertension and type 2 diabetes for many years, and was poorly controlled; myocardium and angiocarpy were severely damaged, so VSR occurred after AMI. In case 1, from the results of enzyme spectrum and troponin, VSR occurred day 2 following AMI. VSR in case 2 occurred the seventh day after AMI. These two cases were in consistent with diagnostic criteria that VSR occurs within 1 week after AMI [1]. All the three VSR cases had history of uncontrolled hypertension, and case 1 and case 3 had history of uncontrolled type 2 diabetes. Hypertension and diabetes are two major factors of cardiovascular disease. Diabetes may cause severe injury of cardiovascular system and myocardium. Clinical studies have confirmed that patients with diabetes suffer from coronary heart disease not only more common, but also with unfavorable prognosis. Pathoglucemia may defunct the preconditioning of myocardial ischemia, resulting in a series of myocardial microvascular and endothellular lesions, such as endothelial dysfunction, and then influence myocardial remodeling and cardiac potential refactoring after AMI. In addition, long-term hypertension leads to left ventricular hypertrophy, expansion and interstitial fibrosis. Hypertension with comorbidity of T2DM aggravates lesions of cardiovascular and myocardium. In case 1 and case 2, VSR occurred immediately after AMI, and the rupture size progressed to 10 mm rapidly, in ethmoid. But in case 3, the patient was younger, she was treated with conservative methods, heart rate was controlled around 60 beats/min, and BP maintained 90/60 mmHg, the intake and output fluid volumes were kept balance, the patient’s condition was stable, and was discharged.

### Treatment of AMI complicated with CR

The high mortality of AMI complicated with CR is mainly attributed to the lack of effective treatment. Presently, the main treatment modalities include conservative medical management, surgery, percutaneous intervention and heart transplantation.

#### Conservative treatment

The aim of conservative management is to maintain the function of circulation and respiration, and keep the vital signs stable. Patients are treated with medicine actively, and undergo intro-aortic balloon pump (IABP) to get prepared for elective surgery. Most patients with this disease have dyspnoea, so oxygen partial pressure and blood oxygen saturation are enhanced by using mask oxygen inhalation, and continuous positive airway pressure.

#### Surgical repair

It is still controversial regarding the surgical repair timing. In the past, it was thought that surgery should be performed 2 - 3 months after VSR, because at that time the edema of myocardium almost completely subsides, the necrotic tissue around the crevasse is fully fibrotic and the patch can stay in place after suturing. However, many clinical trails confirmed that 33% of patients died within the first 24 h after VSR, 70% within first week, and only 15% can survive 1 month for surgery [2]. So now, an earlier surgery is suggested [3]. For VSR patients with rapid deteriorating along with large rupture, the only way is urgent surgery in order to restore circulation dynamics. The satisfactory emergent surgery outcomes are usually from the patients who are positively reacted to IABP, or one’s rupture is in the apex and the surgical route is via left ventricular. On the contrary, if patients do not react to IABP well, or whose rupture is in right ventricular septum, the surgical results will not be ideal.

#### Percutaneous intervention

In recent years, percutaneous VSR umbrella plugging technology was developed and progressed, and this technology is a good choice for patients with early AMI complicated with VSR [4]. Because of its safety and efficacy, it can be a good alternative for patients who are not indicated for surgery.

However, two out of the three cases we reported died 2 - 3 days after VSR, which occurred immediately after AMI, because this left insufficient time interval for surgical repair or percutaneous invention. In addition, given the surgical repair was performed under this circumstance, the fragile heart tissue would not be sutured properly, which might yield unfavorable outcome.

#### Heart transplantation

Heart transplantation is considered the ideal treatment for AMI complicated with VSR. However, due to shortage of donors and high cost, it hardly becomes a conventional treatment.

#### Embryonic stem cell therapy

In recent years, experts began to probe the potential of embryonic stem cell for treatment of AMI with VSR. Under specific induction, embryonic stem cells can differentiate into many types of myocardial cells *in vitro*, including sinoatrial node cell, Purkinje’s cell, atrial muscle cell, ventricle myocytes, and so on, and these differentiated cells might be a potential source of cell transplantation [5]. However, embryonic stem cell therapy still is in trial phase, and the main concerns include transplanted cell survival rate, retention rate after transplantation and the risk of teratoma, immunological rejection.

### Conclusions

Prevention of VSR from occurrence is important, and electrocardiogram, angiography and echocardiography can be of help for diagnosis. When AMI occurs, the early reperfusion therapies, percutaneous coronary intervention and thrombolysis, combined with antiplatelet, heparin or low molecular heparin, statins, can improve patients’ prognosis, and reduce mortality. Since VSR complicating AMI has a high mortality, the early active treatment of primary diseases and revascularization can prevent or reduce its occurrence.
